# From Breast to Orbit: A Case Report of Metastatic Breast Cancer With Orbital Involvement

**DOI:** 10.7759/cureus.66927

**Published:** 2024-08-15

**Authors:** Alhanouf M AlMansour, Nada A Almutairi, Nafisa Abdul Hafiez, Mohammad Alkaiyat

**Affiliations:** 1 Oncology, King Saud Bin Abdulaziz University for Health Sciences College of Medicine, Riyadh, SAU; 2 Oncology, Ministry of National Guard Health Affairs (MNGHA), Riyadh, SAU; 3 Oncology, King Abdulaziz Medical City, Riyadh, SAU

**Keywords:** targeted therapy, treatment, radiotherapy, orbital metastases, infiltrating ductal carcinoma, breast cancer

## Abstract

The approach to manage breast cancer has undergone a significant transformation, leading to longer survival rates. However, there is still a rise in metastasis occurring in less common locations such as the orbit. We report the case of a 40-year-old female diagnosed with luminal A, left-side breast cancer, back in September 2020. She presented with de novo metastatic diseases to the liver, bone, lung, and orbit. She received palliative radiation therapy (RT) to the orbit at the dose of 25 Gray (Gy) in five fractions, and follow-up brain magnetic resonance imaging (MRI) indicated a positive response to treatment with a slight reduction in the size of the left infraorbital lesion. Systemic treatment was started with hormonal therapy fulvestrant and luteinizing hormone-releasing hormone (LHRH), leuprolide, accompanied by palbociclib. As the incidence of ocular metastasis from breast cancer increases, oncologists need to be vigilant about symptoms and use appropriate diagnostic techniques.

## Introduction

Orbital metastases are relatively uncommon, representing 1-13% of all orbital tumors and impacting approximately 2-5% of individuals with systemic malignancies [1-3]. They are frequently caused by breast cancer, followed by prostate cancer, lung cancer, cutaneous melanoma, kidney cancer, and gastrointestinal tract cancer [3-5]. Breast cancer accounts for approximately 30-50% of orbital metastases, with patients exhibiting diverse time intervals between the diagnosis of breast cancer and the onset of ocular metastasis. Within these cases, 40.4% of the patients manifested eye metastasis as the initial presentation of breast cancer, while 59.5% developed ocular metastasis either as the sole site of metastasis or concurrent with the progression of previously diagnosed breast cancer [6]. The purpose of this report is to present the case of orbital metastases as an incidental finding of breast cancer. 

## Case presentation

Patient information, clinical finding, and diagnostic assessment

We report the case of a 40-year-old female who was diagnosed on September 27, 2020, with T1N1M1 (lungs, bone, liver, and orbit), infiltrating ductal carcinoma (IDC) of the left breast, Scarff-Bloom-Richardson (SBR) grade 2/3 (architectural score 3, nuclear grade 2, mitotic score 2), estrogen receptor (ER)/progesterone (PR) positive/human epidermal growth factor receptor 2 (HER-2) negative, and Ki-67 protein of 15%. The patient presented with severe back pain, and the bone scan revealed multiple bone metastases, including the right humerus, inferior scapula, and scapular spine extending to the acromion and the distal clavicle. She underwent genetic testing because of her age, and it showed AKT mutation (somatic), AKT1 c:49G>A (p.E17K), and BRCA gene testing was negative. Laboratory investigations revealed increased tumor marker CA 15-3=157 U/mL (normal ≤30 U/mL).

**Figure 1:**
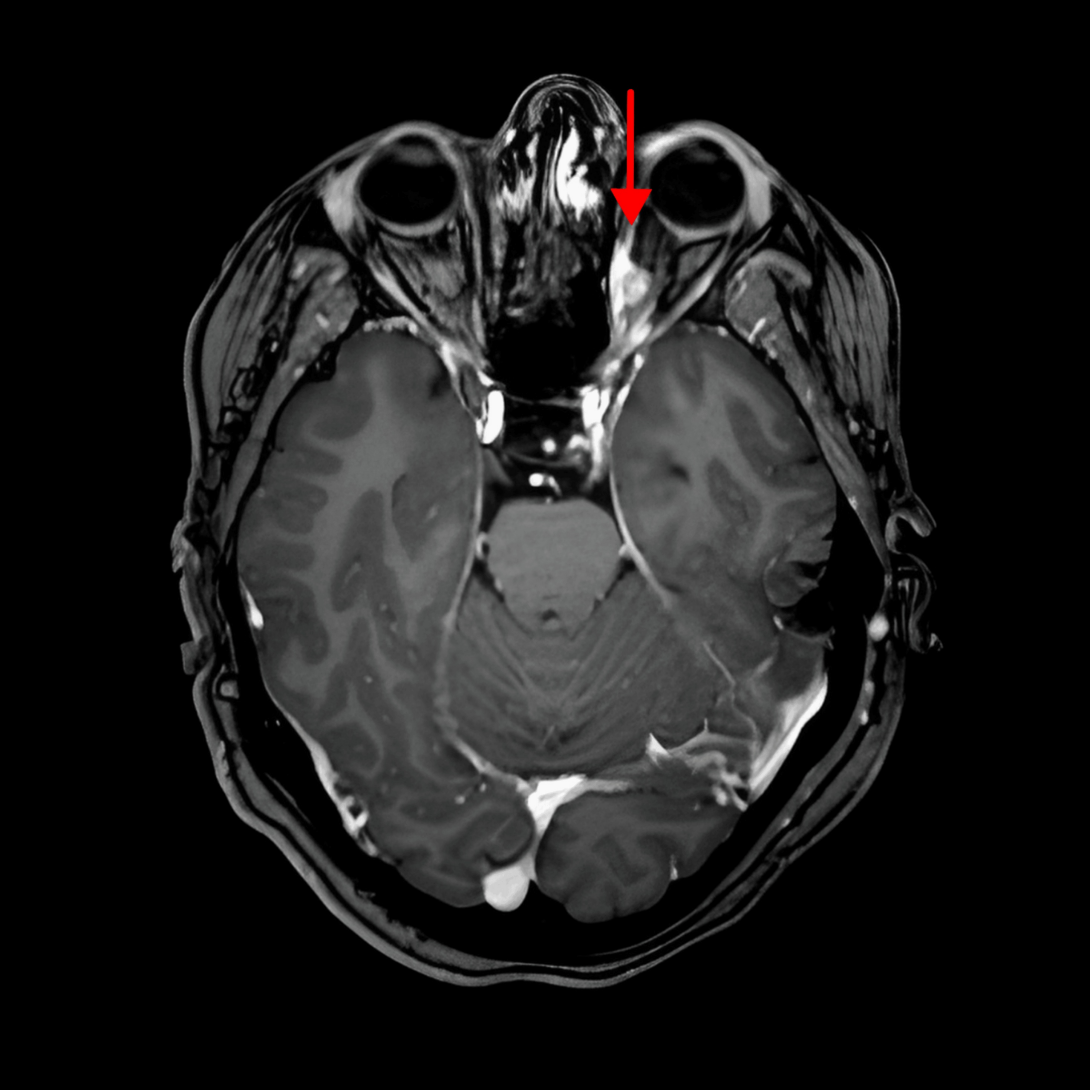
Brain MRI at diagnosis: left intracoronal nodule (9×6 mm) along the medial rectus muscle on the left side MRI: magnetic resonance imaging

**Figure 2:**
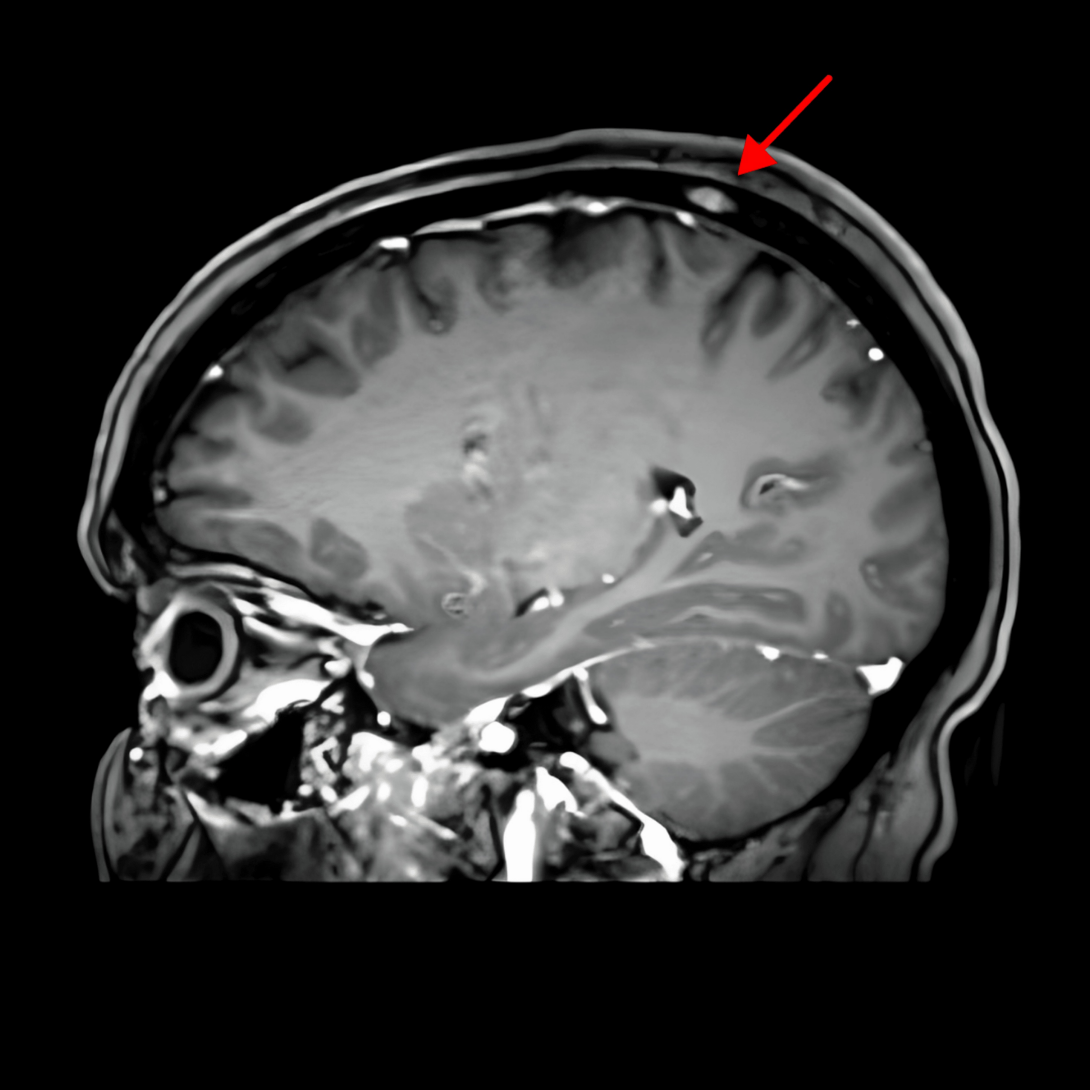
Brain MRI at diagnosis: right parietal bone lesion MRI: magnetic resonance imaging

Treatment was started with hormonal therapy luteinizing hormone-releasing hormone (LHRH) analogs, leuprolide 7.5 mg every four weeks, fulvestrant 500 mg (days 1, 14, and 29) intramuscular and then 500 mg every four weeks, and anti-cyclin-dependent kinase (CDK) 4/6 inhibitor, palbociclib 125 mg orally for 21 days, cycle every 28 days. Our patient was experiencing mild eye pain, which was insignificant, but the oncologist preferred to do brain magnetic resonance imaging (MRI) to rule out brain metastasis. A brain MRI was done on September 27, 2020, and it showed a left intraorbital intracoronal nodule representing orbital metastasis (Figure 1) and a right parietal bone lesion representing skull metastasis (Figure 2). There was no evidence of brain metastasis.

Therapeutic intervention and outcome of treatment

**Figure 3:**
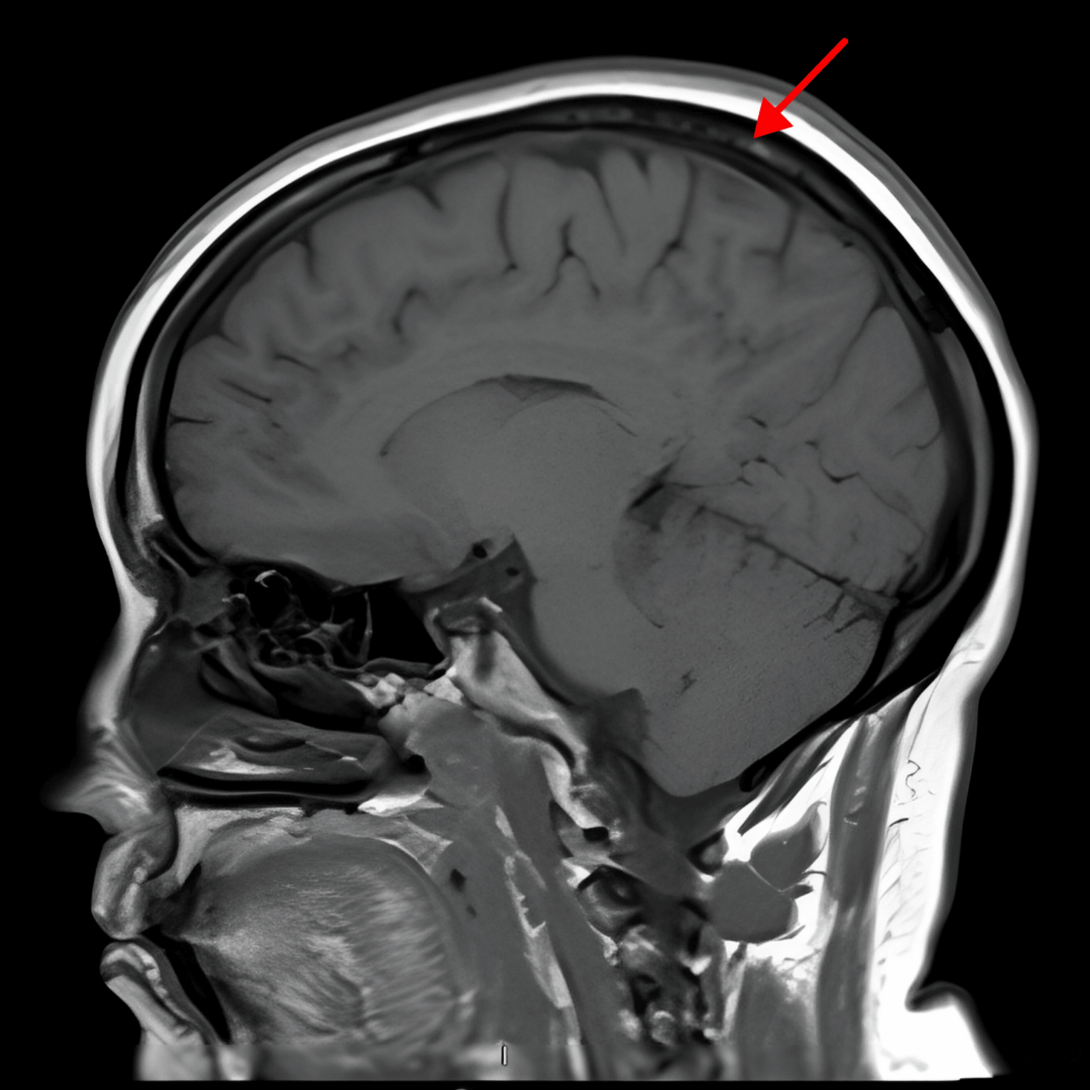
Brain MRI for follow-up post-radiotherapy: right parietal bony lesion shows reduced enhancement with surrounding edema MRI: magnetic resonance imaging

**Figure 4:**
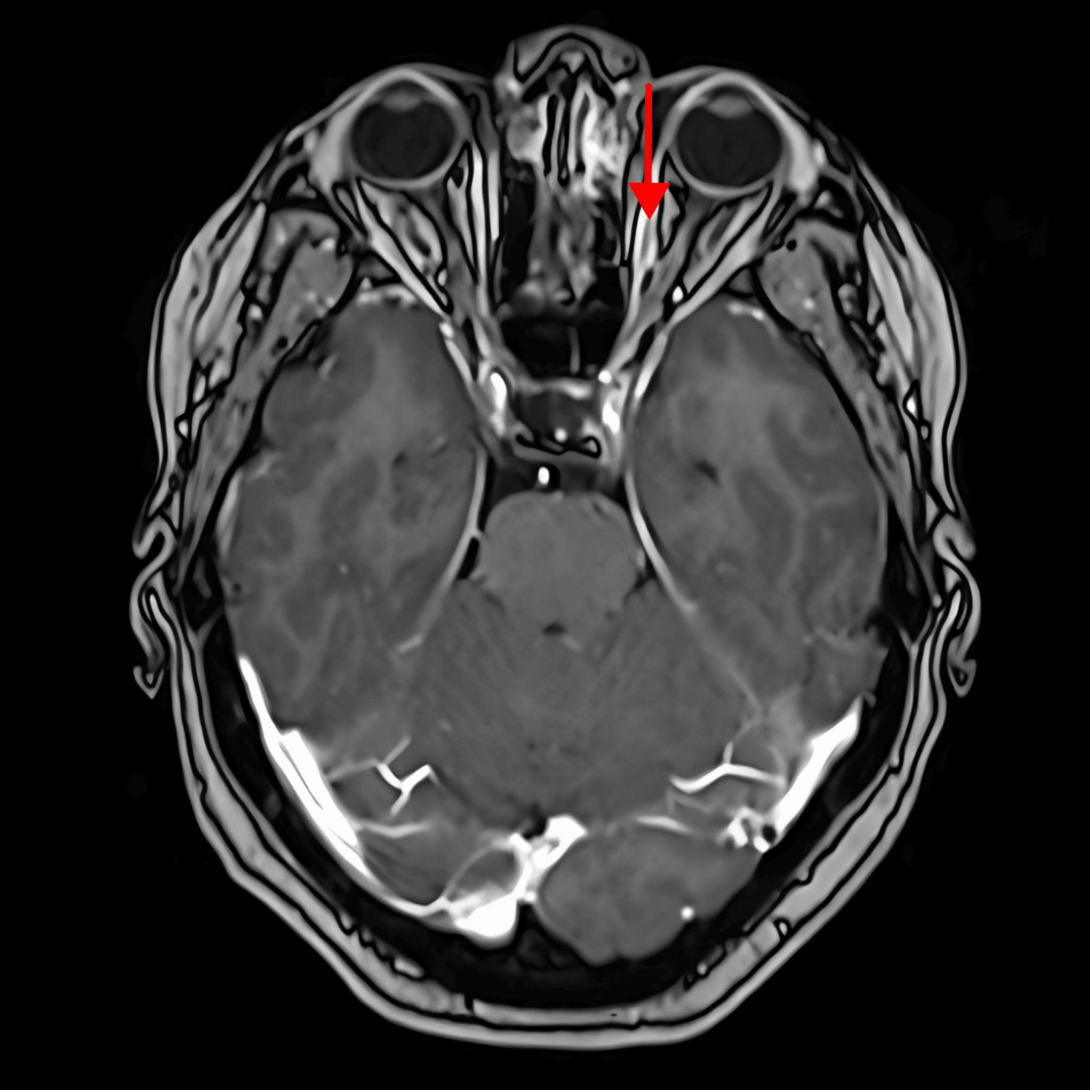
Brain MRI for follow-up post-radiotherapy: reduction in the size of the left intraorbital intracoronal lesion (7×4 mm) MRI: magnetic resonance imaging

After a multidisciplinary discussion, the patient received palliative radiation therapy (RT) to the orbit and skull at a dose of 25 Gray (Gy) in five fractions. A follow-up brain MRI was done on January 7, 2021, and it showed an interval response to the treatment of the right parietal bone lesion (Figure 3) and a slightly reduced size of the left infraorbital lesion (Figure 4).

Abdominal and pelvic computed tomography (CT) showed an interval improvement of metastatic liver lesions, and chest CT showed a significant improvement in lung metastasis. The tumor marker CA 15-3 decreased to 117 U/mL. After dental clearance, the patient started on denosumab 120 mg subcutaneously every four weeks. She was doing great in her last clinic visit which was on May 29, 2024, and her performance status was 1, and she was tolerating fulvestrant, palbociclib, and LHRH very well. Her last follow-up bone scan on November 21, 2023, showed stable heterogeneous radiotracer activity involving the dorsolumbar spine and sternum, with no new lesions identified. The recent CA 15-3 level decreased simultaneously to a normal level (13.7 U/mL), most likely corresponding to the therapeutic effect. Chest and abdominal scans were done on May 6, 2024, revealing stable disease.

## Discussion

Metastatic orbital disease accounts for 1-13% of all orbital tumors, with an average time interval of 2-8.5 years from breast cancer diagnosis to the onset of ocular metastasis [1,7]. In our case, the orbital metastasis was incidentally diagnosed within a month of breast cancer diagnosis after a brain MRI was done to rule out brain metastases. Orbital metastasis can be attributed to the anatomy of the orbit that demonstrates limited lymphatic drainage, suggesting a systemic hematologic dissemination of the distant primary tumor [8]. As observed in our case, which is ER/PR sensitive, most primary tumors that originate from breast cancer and metastasize to the orbits are hormone-sensitive tumors, which can be explained by the cellular tropism caused by estrogen produced by the periorbital fat [7,9]. Although our case was diagnosed with IDC, it was reported that infiltrative lobular carcinoma (ILC) was five times more frequently associated with orbital metastases compared to IDC [10].

Orbital metastasis frequently presents with proptosis (52.3%), relative afferent pupillary defect (RAPD) (38.7%), and diplopia of the ipsilateral eye (35.5%) [11]. However, a retrospective study showed that only 0.7% of orbital metastasis from breast cancer were symptomatic [12]. In our case, the patient was asymptomatic, and the orbital metastasis was an incidental finding. MRI and a contrasted CT are useful diagnostic tools for detecting metastatic lesions; nevertheless, histological examination by fine needle aspiration or open surgical biopsy of the lesion is needed for the definitive diagnosis [13,14]. A systemic review of orbital metastasis demonstrated that incisional biopsy was preferred over fine needle aspiration (63.7% vs. 10.2%), respectively, while partial resection was preferred over complete resection, accounting for 16.6% and 9.5%, respectively [11].

Although there is a wide range of treatment modalities, including surgery, chemotherapy, hormonal therapy, or a combination of these approaches, orbital radiotherapy is the most effective treatment in controlling the size of the tumor and has been reported to significantly improve symptoms compared to not receiving radiotherapy [6,8,9,11,15]. Patients with orbital metastasis were reported to have a poor prognosis, with one-year survival rates highest in breast (57%) and carcinoid (53%) cancers compared to other highly aggressive tumors such as lung and liver carcinomas and malignant melanoma [11].

## Conclusions

Metastatic spread from breast cancer to the orbit is a rare finding that is highly associated with poor prognosis. As orbital metastasis can be asymptomatic, it is highly recommended for healthcare professionals to perform full diagnostic workups at the time of initial diagnoses once the patient is symptomatic in order to detect any orbital metastases and provide appropriate treatment or close monitoring. Additionally, introducing new radiotherapy techniques for treating breast cancer with orbital metastasis can reveal new aspects for improving outcomes. Finally, we think it's crucial to report and share experiences with these cases due to the limited data available in this area.
